# Liver Governs Tendon: A Theory from Traditional Chinese Medicine—Evidence from a Population-Based Matched Cohort Study in Taiwan for the Association of Chronic Liver Disease and Common Diseases in the Chiropractic Office

**DOI:** 10.1155/2016/7210705

**Published:** 2016-06-29

**Authors:** Chia-Man Ma, Lih-Hwa Lin, Yung-Hsiang Chen, Huey-Yi Chen, Jen-Huai Chiang, Wen-Chi Chen

**Affiliations:** ^1^Department of Dermatology, Taichung Veterans General Hospital, Taichung 407, Taiwan; ^2^Division of Chinese Medicine, China Medical University-An Nan Hospital, Tainan 709, Taiwan; ^3^Graduate Institute of Chinese Medicine, School of Chinese Medicine, Graduate Institute of Integrated Medicine, Research Center for Chinese Medicine & Acupuncture, China Medical University, Taichung 404, Taiwan; ^4^Departments of Medical Research, Obstetrics and Gynecology, and Urology, Management Office for Health Data, China Medical University Hospital, Taichung 404, Taiwan; ^5^Department of Psychology, College of Medical and Health Science, Asia University, Taichung 413, Taiwan

## Abstract

In traditional Chinese medicine (TCM) theory, the liver governs the tendons. This retrospective cohort study investigated the relationship between chronic liver disease and common orthopedic conditions by utilizing the National Health Insurance Research Database of Taiwan. The populations included within this study were chronic liver disease patients (International Classification of Diseases/ICD-9 code: 571) and a comparison group composed of patients with nonchronic liver disease. The medical event that was evaluated was internal derangement of joints (ICD-9 codes: 717-718). In comparison with the control group, patients with chronic liver disease were 1.29 times more likely to develop internal derangement of joints when major trauma had also occurred. We did not find the association of viral hepatitis with internal derangement of joints. Patients with chronic liver disease as well as anemia were 3.01 times more likely to develop joint derangements. Our study shows that patients with anemia in addition to chronic liver disease are more prone to develop joint derangements. This is the first documented research study that endorses “the liver governs the tendons which gives the body the ability to move” theory of TCM. The incidence rate of internal derangement of knee joints was higher in patients with chronic liver disease.

## 1. Introduction

Chronic liver disease is a common disease in Taiwan, and according to an official report from the Taiwanese government in 2011, hepatoma affected 25/100,000 men and 10/100,000 women [1–3]. The incidence of hepatitis type B virus in Taiwan was 13.18%. In 2009, the incidence of hepatitis type B was 15.85% in men and 11.06% in women. There is a significant relationship between hepatitis and hepatomas in Taiwan. The World Health Organization estimates that 240 million people, about 3.7% of the world's population, are infected with chronic hepatitis B [4, 5], which means that chronic hepatitis B infection is also a major global health problem [6, 7]. Hepatitis C virus (HCV), the major cause of chronic liver disease and liver transplantation, has a global prevalence of 3%, with 170 million people infected worldwide [8]. However, the relationship between chronic liver disease and chiropractic disease is unknown and is not well documented.

A basic concept of Traditional Chinese Medicine (TCM) states that there are five important endoorgans that govern other organs or tissues, and these five organs are the liver, heart, spleen, lung, and kidney. This concept states that the liver governs the tendons which give the body the ability to move. Consequently, dysfunction of the liver leads to impairment of body movements. Some of these impairments may be related to the internal derangement of joints. Following this principle, we investigated the correlation between chronic liver disease and internal joint derangements, a common disease in the chiropractic field. This study was based on a nationwide, population-based cohort study in Taiwan.

## 2. Materials and Methods

### 2.1. Database

This is a retrospective cohort study. Claimed data from the National Health Insurance Research Database (NHIRD) was used in this study (LHID 2000). Data was obtained from health care data of >96% of all medical claims in Taiwan since 1996. Medical services provided by the NHI Program included both Western and Traditional Chinese Medicine (TCM) of outpatient care, inpatient care, physical therapy, dental services, prescription drugs, medical institutions, and registration files with scrambled identifications [9].

Representative data between 2000 and 2011 was randomly obtained from the claims dataset of a sample of one million enrolled from the entire insured population in the NHI Program database (database: LHID 2000). The diagnoses code was used from the International Classification of Diseases 9th Revision of the Clinical Modification (ICD-9-CM) in the database. Detailed description of the NHIRD was previously described [10].

The study population was newly diagnosed chronic liver disease patients (ICD-9 code: 571) between January 1, 2000, and December 31, 2011, as case cohort. Additional analyses by subgroup of chronic liver disease included chronic hepatitis (ICD-9: 571.4, excluding HBV and HCV), alcoholic hepatitis (ICD-9-CM: 571.0, 571.1, 571.2, and 571.3), hepatitis B virus (HBV) (ICD-9-CM: 070.2, 070.3, and V02.61), and HCV (ICD-9-CM: V02.62, 070.41, 070.44, 070.51, 070.7, and 070.54) which were further analyzed in the study population.

The comparison cohort was patients with nonchronic liver disease (control group). In the control group, four controls were frequency matched with age (every 5 years), gender, and index year. The age was >18 in both groups. The event was internal derangement of joints (ICD-9 codes: 717-718) which represented difficulty in the motion of joints, that is, due to lack of governing by liver in the principle of TCM. The patients were counted until end of follow-up at 2011/12/31.

Patients having internal derangement of joints (ICD-9 codes: 717-718) and any cancers (ICD-9 codes: 140–208) before diagnosis of chronic liver disease were excluded. We also excluded diseases associated with skeletal system that may cause progress of joint derangement naturally which may be a confounder, such as congenital bone disease (ICD-9 codes: 754, 755, and 756), diseases of the musculoskeletal system and connective tissue (ICD-9 codes: 710–716, 719, 730, 733, and 736), fracture of patella (ICD-9 code: 808), fracture of lower limb (ICD-9 codes: 820, 821, 822, and 823), any dislocations (ICD-9 codes: 835-836), sprains and strains of knee and leg (ICD-9 code: 844), and late effects of musculoskeletal and connective tissue injuries (ICD-9 codes: 905–909). Major trauma (ICD-9 codes: 800–999) and anemic disorders (ICD-9 codes: 280–285) were listed as comorbidity.

### 2.2. Statistical Analyses

A chi-square test was carried out to examine the differences in the distribution of sociodemographic factors and comorbidities between the cohorts and control group. A Cox regression model was used to examine the incidence of following internal derangement of joints and 95% confidence interval (95% CI) of categorical variables was calculated for each cohort. Multivariate Cox proportional hazard models were used to estimate hazard ratio (HR) and 95% CI for factors associated with comorbidity such as age, gender, and comorbidities. Whole analysis was performed by using the SAS statistical package (SAS System for Windows, Version 9.4) with the statistical adopted significance level at 0.05.

## 3. Results

We identified a total of 19,776 patients coded with chronic liver disease and 79,104 patients, 4 times the number of coded patients, as the control group. The demographic data are listed in Table 1. The gender distributions were equal in both groups, with the male gender representing 67.8% of each group. The age distribution was the same across the two groups. Patients who were between 18 and 39 years old comprised 61.18% of the study population, while patients aged between 40 and 64 years old comprised 35.73% and those aged over 65 years comprised 3.09% of the study population. The mean age of the control group was 37.26 ± 12.82 years, and the mean age was 37.64 ± 12.56 years in patients with chronic liver disease. Both groups suffered from major trauma: 70.37% of the patients without chronic liver disease and 78.84% of those with chronic liver disease. Regarding anemia, 3.45% of patients without chronic liver disease and 7.78% of patients with chronic liver disease were anemic. There were 125 (0.63%) patients with chronic liver disease who also had internal derangement of joints listed in the data bank, compared to the control group which consisted of 357 patients (0.45%). Patients with chronic liver disease had a significantly higher statistical probability to be affected by internal derangement of joints. In chronic liver disease group, there were 17,421 chronic hepatitis cases: 4,637 patients with hepatitis B, 1,008 patients with hepatitis C, 401 patients with acute alcoholic hepatitis, and 527 patients with alcoholic cirrhosis of liver.

A Cox regression model analysis yielded hazard ratios (HRs) and 95% CIs of internal derangement of joints associated with chronic liver disease and covariates, as listed in Table 2. The hazard ratio of chronic liver disease associated with internal derangement of joints was 1.26 (95% CI = 1.05–1.54, *p* = 0.0288) in a Cox model regression analysis. Comorbidities included major trauma and anemia and both revealed significant HR values (3.41, 95% CI = 2.52–4.62, *p* < 0.001, and HR 1.62, 95% CI = 1.13–2.31, *p* = 0.0081, resp.).

In Table 3, we classified data by demographic factors in order to analyze the incidence rates and HRs of internal derangement of knees associated with chronic liver disease. The female gender group showed a higher hazard ratio of 1.53 and a 95% CI = 1.07–2.18. If major trauma or anemic disorder was present as a comorbidity, higher hazard ratios were observed as follows: 1.26 and 2.80, respectively. There was HR of 1.03 (95% CI = 0.69–1.55) in the group of HBV (Table 4). There was HR of 1.21 (95% CI = 0.54–2.71) in the group of HCV (Table 5).  Table 6 describes the HR of acute alcoholic hepatitis of 1.03 (95% CI = 0.26–4.15). By calculating, grouping another chronic hepatitis coded as 571.4, the HR was 1.3 (95% CI = 1.05–1.60) which was significant difference with control group (Table 7, *p* < 0.05).

We used the Cox proportional hazard regression analysis to compare the risk of internal derangement of knee-associated chronic liver disease with major trauma and anemia (Table 8). Both patients with or without chronic liver disease had higher hazard ratios if major trauma was present. Hazard ratios and 95% CIs were 4.30 (2.98–6.21) and 3.42 (2.44–4.79), respectively. Only patients with chronic liver disease and anemia as their comorbidity had significant statistics of HR 2.90, 95% CI = 1.87–4.49.

## 4. Discussion

In our study, the incidence rates of internal derangement of knees were higher in patients with chronic liver disease, especially when major trauma or anemia was also present. This result corresponds with the principle “the liver governs the tendons which give the body the ability to move,” a theory of TCM [11, 12]. However, there was no significant difference in the groups of viral hepatitis B and C.

Clinical studies about arthritis associated with liver disease have already been well established for many years. The possible association between arthritis and liver disease was first observed in 1897 by Still and in 1903 by Weber [13]. Currently, it is apparent that symptoms of joint problems could be important indicators of the systemic nature of many liver diseases [14]. Arthritis may occur in association with a variety of liver diseases [15–17]. After the excellent review of arthropathy and liver disease published by Whelton in 1972, the amount of research concerning systemic manifestations of liver or joint diseases increased [18]. A case of active chronic hepatitis complicated by small joint arthropathy was reported in 1973 [19]. The striking symptoms of the arthritis in this case were the visible soft tissue swelling and gross bone destruction occurring within the joints, which were completely painless and retained full range of motion. Radiographically proven bone erosions and joint changes were consistent with rheumatoid arthritis [18–20]. Interestingly, the frequency with which antiglobulins are present in the serum of patients with active hepatocellular disease greatly reduces the diagnostic value of serological tests for rheumatoid factors [21]. In 1978, 83 patients with primary biliary cirrhosis were investigated to determine the prevalence of rheumatic disorders. A high prevalence of scleroderma, which often affects joint movements, was examined [22].

Some studies indicated that the increase in gamma globulins or decreased serum levels of immune complexes may play an important role in the relationship between chronic liver disease and arthritis. A clinical study of young women with a severe form of hepatic cirrhosis was published in 1956. Symptoms such as arthritis, obscure febrile episodes, and occasional hormonal disturbances were observed. Laboratory results showed that acute stages of the disease usually revealed an extremely increased amount of serum gamma globulins and an increase in the number of plasma cells of the liver [23]. In 1971, transient polyarthritis, which presented as the prodromal phase of acute viral hepatitis, was reported and was thought to be related to immune system complexes [24]. A correlation between low serum levels of complement in viral hepatitis and high titers of hepatitis-associated antigen was observed. Serum-sickness-like syndrome, caused by circulating immune system complexes [25, 26], was believed to be correlated with skin rashes and joint problems, along with hepatitis-associated antigens. This condition showed low levels of complement activity during the acute state. These previous reports prompted us to investigate the relationships between chronic liver diseases, anemia, and joint derangements.

A trend showing joint derangements in patients with chronic liver disease was observed in our research, especially when major trauma or anemia was a comorbidity. In addition, results from the female group were more apparent than those from the male group. There are many different hypotheses regarding the pathogenic mechanisms of chronic liver disease and arthritis in modern Western medicine. Some authors suggest that arthritis and HCV infection coexist by coincidence. Other authors believed that HCV might act as a trigger for other diseases in genetically predisposed individuals. This hypothesis states that HCV directly invades synovial cells possibly through a local inflammatory response, cytokine-induced disease, or diseases involving immune complexes [27]. Lastly, some authors surmised that HCV causes a distinct infectious arthritis. An HCV-related arthritis that responded to therapy with interferon alpha has been reported. In this study, we did not find the association of viral hepatitis with internal derangement of joints. Therefore, we cannot fully understand the mechanisms involved in the response to therapy [28].

According to our research data, patients with chronic liver disease are 1.29 times more likely to develop internal derangement of joints when major trauma is present. Patients with chronic liver disease and anemia were 3.01 times more likely to develop internal derangement of joints. It is fascinating that when we make comparisons between major trauma and anemia, anemia is more likely to cause patients with chronic liver disease to develop joint derangements. There are similar concepts between anemia and chronic liver disease in modern Western medicine that are already known and accepted. Thrombocytopenia, which is known to occur in patients with chronic hepatitis C, is a hematological condition defined as a reduced blood platelet count. The pathophysiology of thrombocytopenia in patients with HCV infection is still incompletely understood, despite intensive studies performed over decades, but it is believed to be influenced by multiple factors which influence the severity of the disease [29]. Currently, there are many proposed causes of thrombocytopenia including hypersplenism, antiplatelet antibody formation, or decreased thrombopoietin production [30]. In other reports, HCV is thought to be an etiological agent, in which the replication and expression of viral proteins in extrahepatic tissues contribute to extrahepatic manifestations, such as mixed cryoglobulinemia or arthritis [31].

We compared the separate relationships between chronic liver diseases with two comorbidities, major trauma and anemia. Patients with or without chronic liver disease and with major trauma as comorbidity had a higher risk of developing internal derangement of the knees. It can be deduced that joint derangements could be a complication after major trauma. Additionally, we found that patients with chronic liver disease and major trauma had the highest risk of suffering from internal derangement of the knees, with a hazard ratio of 4.30, and patients with major trauma without liver disease have a ratio of 3.42. Therefore, chronic liver disease plays an important role in the development of internal derangement of the knees. There are two explanations for this: first, patients with chronic liver disease developed more severe symptoms when they encountered a major trauma. It could be the poor quality of tendons, caused by chronic liver disease, that lead to the development of a more severe injury after a major trauma, as explained in TCM theory. Secondly, internal derangement of the knees is not always caused by major trauma. Sometimes, chronic liver disease could be the result of tendon dysfunction, which may lead to joint derangements. Patients with chronic liver disease and anemia as well revealed a significant statistic which means 2.9 times the control group. According to the traditional concept of TCM, sick tendons caused by chronic liver disease worsen if anemia is present, because blood flow and nutrition of tendons are affected. Our results are compatible with modern Western medicine opinions that claim that arthritis and low levels of complement activity in acute state are sometimes manifestations of chronic liver disease.

Our study has made some stronger points when compared to previous studies. The source of our database is a heterogeneous, nationwide database which was obtained from different hospitals, medical centers, and clinics. The study population is large and was obtained from 2000 to 2011. In addition, diseases associated with the skeletal system that may cause the progression of joint derangement eventually were excluded. For patients over 65 years of age, who comprised 3.09% of the study population, it can be assumed that their joint problems are almost always caused by age-related arthritis instead of liver disease. In conclusion, our study is more reliable because of these statistics.

However, there are several limitations of this cohort study. We were not able to obtain the severity of the major trauma and we lacked data for the trauma aspects of the patient history. Secondly, we did not record the interval between the developments of joint derangements after the diagnosis of chronic liver disease and after one of the comorbidities began.

According to TCM, there are five important viscera: liver, heart, spleen, lungs, and kidneys. Each organ has its own corresponding internal organs. In Huang Di Nei Jing (Yellow Emperor's Canon of Internal Classic, *黃帝內*經), the liver governs the tendons which give the body the ability to move [32]. Our results support this principle, which is also compatible with modern Western medicine opinions. In this case, regular follow-up for joint derangement in patients with chronic liver disease is recommended, especially when major trauma or anemia is also present. If joint pain is experienced by a patient, further aggressive evaluations, such as radiographic examination or a consultation with a rheumatologist, are recommended. In other words, patients with rheumatic disorders who have abnormal liver function or hepatomegaly need further hepatic investigations, such as abdominal sonography, or liver biopsy if needed [17]. Further studies should evaluate if treating of liver disease or correcting anemia can prevent joint disease progression.

## 5. Conclusions

To the best of our knowledge, this is the first study to support “the liver governs the tendons which give the body the ability to move” theory of TCM. The results revealed that the incidence of internal derangement of the knees is higher in patients with chronic liver disease, especially when major trauma or anemia is present as a comorbidity.

## Figures and Tables

**Table 1:** Demographic characteristics and comorbidity in patients with and without chronic liver disease.

Variables	Chronic liver disease				*p* value
	No		Yes		
	(*N* = 79104)		(*N* = 19776)		
	*n*	%	*n*	%	
*Sex*					
Female	25472	32.2	6368	32.2	0.99
Male	53632	67.8	13408	67.8	
*Age, years *					
18–39	48396	61.18	12099	61.18	0.99
40–64	28260	35.73	7065	35.73	
More than 65	2448	3.09	612	3.09	
Mean (SD)^†^	37.26 ± 12.82		37.64 ± 12.56		0.0002
*Baseline comorbidity*					
Major trauma	55668	70.37	15592	78.84	<0.0001
Anemic disorder	2732	3.45	1538	7.78	<0.0001
HBV	0	0	4637	—	—
HCV	0	0	1008	—	—
Acute alcoholic hepatitis (ICD-9-CM: 571.1)	0	0	401	—	—
Alcoholic cirrhosis of liver (ICD-9-CM: 571.2)	0	0	527	—	—
Chronic hepatitis (ICD-9-CM: 571.4)	0	0	17421	—	—
*Outcome*					
Internal derangement of knee	357	0.45	125	0.63	0.0011

Chi-square test; ^†^two-sample *t*-test.

**Table 2:** Cox model measured hazard ratio and 95% confidence intervals of internal derangement of knee associated with chronic liver disease and covariates.

Characteristics	Crude			Adjusted		
	HR	(95% CI)	*p* value	HR	(95% CI)	*p* value
*Chronic liver disease (ref = no)*						
Yes	1.38	(1.13–1.69)	0.0018	1.26	(1.02–1.54)	0.0288
*Gender (ref = male)*						
Female	0.92	(0.75–1.11)	0.3679	0.88	(0.72–1.07)	0.206
*Age, years (ref = 18–39)*						
40–64	0.99	(0.82–1.20)	0.9466	1.06	(0.87–1.28)	0.577
More than 65	1.23	(0.72–2.10)	0.4534	1.29	(0.76–2.21)	0.3496
*Comorbidity*						
*Major trauma (ref = no)*						
Yes	3.47	(2.57–4.69)	<0.0001	3.41	(2.52–4.62)	<0.0001
*Anemic disorder (ref = no)*						
Yes	1.70	(1.21–2.40)	0.0025	1.62	(1.13–2.31)	0.0081

HR: hazard ratio; CI: confidence interval.

Adjusted HR: adjusted for age, gender, and comorbidity in Cox proportional hazards regression.

**Table 3:** Incidence rates and hazard ratio for internal derangement of knee to chronic liver disease stratified by demographic factors.

Variables	Chronic liver disease						Crude HR	Adjusted HR
	No			Yes				
	(*N* = 79104)			(*N* = 19776)				
	Event	Person-years	IR	Event	Person-years	IR	(95% CI)	(95% CI)
*Total*	357	491080	0.73	125	124335	1.01	1.38 (1.13–1.69)^*∗∗*^	1.26 (1.02–1.54)^*∗*^
*Sex*								
Female	104	159884	0.65	44	40611	1.08	1.66 (1.17–2.37)^*∗∗*^	1.53 (1.07–2.18)^*∗*^
Male	253	331196	0.76	81	83724	0.97	1.27 (0.99–1.63)	1.14 (0.89–1.47)
*Age, years *								
18–39	233	316173	0.74	78	81066	0.96	1.30 (1.01–1.69)^*∗*^	1.22 (0.95–1.58)
40–64	115	162922	0.71	42	40432	1.04	1.47 (1.03–2.09)^*∗*^	1.29 (0.90–1.83)
More than 65	9	11985	0.75	5	2837	1.76	2.35 (0.79–7.01)	1.74 (0.57–5.29)
*Major trauma*	319	348970	0.91	116	98409	1.18	1.29 (1.04–1.59)^*∗*^	1.26 (1.02–1.56)^*∗*^
*Anemic disorder*	13	17316	0.75	22	9713	2.26	3.01 (1.52–5.97)^*∗∗*^	2.80 (1.40–5.59)^*∗∗*^

IR: incidence rates, per 1,000 person-years; HR: hazard ratio; CI: confidence interval.

Adjusted HR: mutually adjusted for age, gender, and comorbidities in Cox proportional hazards regression.

^*∗*^ *p* < 0.05;  ^*∗∗*^ *p* < 0.01.

**Table 4:** Incidence rates and hazard ratio for internal derangement of knee to HBV stratified by demographic factors.

Variables	HBV						Crude HR	Adjusted HR
	No			Yes				
	(*N* = 79104)			(*N* = 4637)				
	Event	Person-years	IR	Event	Person-years	IR	(95% CI)	(95% CI)
*Total*	351	491356	0.71	25	30747	0.81	1.13 (0.76–1.7)	1.03 (0.69–1.55)
*Sex*								
Female	103	159678	0.65	7	9709	0.72	1.1 (0.51–2.38)	1.01 (0.47–2.19)
Male	248	331677	0.75	18	21038	0.86	1.14 (0.71–1.84)	1.04 (0.64–1.67)
*Age, years *								
18–39	233	316611	0.74	20	22038	0.91	1.23 (0.78–1.94)	1.15 (0.73–1.82)
40–64	105	162879	0.64	5	8353	0.60	0.93 (0.38–2.27)	0.8 (0.33–1.97)
More than 65	13	11865	1.10	0	355	0.00	—	—
*Major trauma*	321	349749	0.92	24	24377	0.98	1.07 (0.71–1.62)	1.07 (0.7–1.61)
*Anemic disorder*	16	17101	0.94	4	2273	1.76	1.86 (0.62–5.56)	1.95 (0.64–5.89)

IR: incidence rates, per 1,000 person-years; HR: hazard ratio; CI: confidence interval.

Adjusted HR: mutually adjusted for age, gender, and comorbidities in Cox proportional hazards regression.

**Table 5:** Incidence rates and hazard ratio for internal derangement of knee to HCV stratified by demographic factors.

Variables	HCV						Crude HR	Adjusted HR
	No			Yes				
	(*N* = 79104)			(*N* = 1008)				
	Event	Person-years	IR	Event	Person-years	IR	(95% CI)	(95% CI)
*Total*	351	491356	0.71	6	6117	0.98	1.38 (0.62–3.09)	1.21 (0.54–2.71)
*Sex*								
Female	103	159678	0.65	1	1849	0.54	0.85 (0.12–6.06)	0.75 (0.1–5.37)
Male	248	331677	0.75	5	4268	1.17	1.57 (0.65–3.81)	1.35 (0.56–3.3)
*Age, years *								
18–39	233	316611	0.74	3	3074	0.98	1.33 (0.43–4.16)	1.22 (0.39–3.8)
40–64	105	162879	0.64	3	2779	1.08	1.68 (0.53–5.29)	1.42 (0.45–4.5)
More than 65	13	11865	1.10	0	263	0.00	—	—
*Major trauma*	321	349749	0.92	4	5018	0.80	0.87 (0.33–2.34)	0.85 (0.32–2.28)
*Anemic disorder*	16	17101	0.94	0	676	0.00	—	—

IR: incidence rates, per 1,000 person-years; HR: hazard ratio; CI: confidence interval.

Adjusted HR: mutually adjusted for age, gender, and comorbidities in Cox proportional hazards regression.

**Table 6:** Incidence rates and hazard ratio for internal derangement of knee to acute alcoholic hepatitis (ICD-9-CM: 571.1) stratified by demographic factors.

Variables	Acute alcoholic hepatitis						Crude HR	Adjusted HR
	No			Yes				
	(*N* = 79104)			(*N* = 401)				
	Event	Person-years	IR	Event	Person-years	IR	(95% CI)	(95% CI)
*Total*	351	491356	0.71	2	2205	0.91	1.28 (0.32–5.14)	1.03 (0.26–4.15)
*Sex*								
Female	103	159678	0.65	0	251	0.00	—	—
Male	248	331677	0.75	2	1954	1.02	1.37 (0.34–5.52)	1.1 (0.27–4.45)
*Age, years *								
18–39	233	316611	0.74	1	1383	0.72	0.99 (0.14–7.06)	0.83 (0.12–5.9)
40–64	105	162879	0.64	1	803	1.25	1.94 (0.27–13.92)	1.62 (0.23–11.64)
More than 65	13	11865	1.10	0	19	0.00	—	—
*Major trauma*	321	349749	0.92	2	1961	1.02	1.12 (0.28–4.49)	1.07 (0.27–4.32)
*Anemic disorder*	16	17101	0.94	0	233	0.00	—	—

IR: incidence rates, per 1,000 person-years; HR: hazard ratio; CI: confidence interval.

Adjusted HR: mutually adjusted for age, gender, and comorbidities in Cox proportional hazards regression.

**Table 7:** Incidence rates and hazard ratio for internal derangement of knee to chronic hepatitis (ICD-9-CM: 571.4) stratified by demographic factors.

Variables	Chronic hepatitis						Crude HR	Adjusted HR
	No			Yes				
	(*N* = 79104)			(*N* = 17421)				
	Event	Person-years	IR	Event	Person-years	IR	(95% CI)	(95% CI)
*Total*	351	491356	0.71	115	111679	1.03	1.44 (1.17–1.78)^*∗∗∗*^	1.3 (1.05–1.60)^*∗*^
*Sex*								
Female	103	159678	0.65	39	36969	1.05	1.63 (1.13–2.36)^*∗∗*^	1.48 (1.02–2.15)^*∗*^
Male	248	331677	0.75	76	74709	1.02	1.36 (1.05–1.76)^*∗*^	1.21 (0.93–1.57)
*Age, years *								
18–39	233	316611	0.74	72	73583	0.98	1.33 (1.02–1.73)^*∗*^	1.24 (0.95–1.61)
40–64	105	162879	0.64	39	35647	1.09	1.7 (1.17–2.45)^*∗∗*^	1.47 (1.01–2.12)^*∗*^
More than 65	13	11865	1.10	4	2448	1.63	1.5 (0.49–4.6)	1.04 (0.33–3.23)
*Major trauma*	321	349749	0.92	108	88406	1.22	1.33 (1.07–1.65)^*∗*^	1.3 (1.04–1.61)^*∗*^
*Anemic disorder*	16	17101	0.94	108	88406	1.22	2.45 (1.27–4.74)^*∗∗*^	2.32 (1.2–4.5)^*∗*^

IR: incidence rates, per 1,000 person-years; HR: hazard ratio; CI: confidence interval.

Adjusted HR: mutually adjusted for age, gender, and comorbidities in Cox proportional hazards regression.

^*∗*^ *p* < 0.05; ^*∗∗*^ *p* < 0.01; ^*∗∗∗*^ *p* < 0.001.

**Table 8:** Cox proportional hazard regression analysis for the risk of internal derangement of knee-associated chronic liver disease with comorbidity.

Variables		Event	Person-years	Rate	Adjusted HR
					(95% CI)
Chronic liver disease	Major trauma				
No	No	38	142110	0.27	1 (reference)
No	Yes	319	348970	0.91	3.42 (2.44–4.79)^*∗∗∗*^
Yes	No	9	25927	0.35	1.27 (0.62–2.63)
Yes	Yes	116	98409	1.18	4.30 (2.98–6.21)^*∗∗∗*^

Chronic liver disease	Anemic disorder				
No	No	344	473764	0.73	1 (reference)
No	Yes	13	17316	0.75	1.03 (0.59–1.81)
Yes	No	103	114622	0.90	1.15 (0.93–1.44)
Yes	Yes	22	9713.5	2.26	2.90 (1.87–4.49)^*∗∗∗*^

Adjusted HR: mutually adjusted for age, gender, and comorbidities in Cox proportional hazards regression.

^*∗∗∗*^ *p* < 0.001.

## References

[1] Chien C.-C., Wang J.-J., Sun Y.-M. (2012). Long-term survival and predictors for mortality among dialysis patients in an endemic area for chronic liver disease: A National Cohort Study in Taiwan. *BMC Nephrology*.

[2] Del Prete A., Scalera A., Iadevaia M. D. (2012). Herbal products: benefits, limits, and applications in chronic liver disease. *Evidence-Based Complementary and Alternative Medicine*.

[3] Jang E., Kim B.-J., Lee K.-T., Inn K.-S., Lee J.-H. (2015). A survey of therapeutic effects of *Artemisia capillaris* in liver diseases. *Evidence-Based Complementary and Alternative Medicine*.

[4] Wu C., Huang H., Cho D. (2015). An acute bleeding metastatic spinal tumor from HCC causes an acute onset of cauda equina syndrome. *BioMedicine*.

[5] Yao H.-T., Yang Y.-C., Chang C.-H., Yang H.-T., Yin M.-C. (2015). Protective effects of (−)-epigallocatechin-3-gallate against acetaminophen-induced liver injury in rats). *BioMedicine*.

[6] Byrne D. D., Newcomb C. W., Carbonari D. M. (2014). Prevalence of diagnosed chronic hepatitis B infection among U.S. Medicaid enrollees, 2000–2007. *Annals of Epidemiology*.

[7] Ott J. J., Stevens G. A., Groeger J., Wiersma S. T. (2012). Global epidemiology of hepatitis B virus infection: new estimates of age-specific HBsAg seroprevalence and endemicity. *Vaccine*.

[8] Kamal S. M. (2008). Acute hepatitis C: a systematic review. *American Journal of Gastroenterology*.

[9] Lu J.-F. R., Hsiao W. C. (2003). Does universal health insurance make health care unaffordable? Lessons from Taiwan. *Health Affairs*.

[10] Tsai K.-S., Yen C.-S., Wu P.-Y. (2015). Traditional Chinese Medicine decreases the stroke risk of systemic corticosteroid treatment in dermatitis: a nationwide population-based study. *Evidence-Based Complementary and Alternative Medicine*.

[11] Zhou W., Benharash P. (2014). Significance of ‘Deqi’ response in acupuncture treatment: myth or reality. *JAMS Journal of Acupuncture and Meridian Studies*.

[12] Hao J., Zhu J., Zhang P. (2015). Our viewpoints on Deqi in the later ages after birth of classical works ‘The Yellow Emperor's Internal Classic’ and ‘Canon of Difficult Medical Problems’. *Zhen Ci Yan Jiu*.

[13] Weber F. P. (1903). A case of the form of chronic joint disease in children described by still. *British Medical Journal*.

[14] Mills P. R., Sturrock R. D. (1982). Clinical associations between arthritis and liver disease. *Annals of the Rheumatic Diseases*.

[15] Mirise R. T., Kitridou R. C. (1979). Arthritis and hepatitis. *Western Journal of Medicine*.

[16] Schumacher H. R., Gall E. P. (1974). Arthritis in acute hepatitis and chronic active hepatitis. Pathology of the synovial membrane with evidence for the presence of Australia antigen in synovial membranes. *The American Journal of Medicine*.

[17] Hilton A. M., Boyes B. E., Smith P. J., Sharp J., Dymock I. W. (1974). Liver disease in patients with joint symptoms. *Annals of the Rheumatic Diseases*.

[18] Whelton M. J. (1972). Arthropathy and liver disease. *Journal of the Irish Medical Association*.

[19] Barnardo D. E., Vernon Roberts B., Currey H. L. F. (1973). A case of active chronic hepatitis with painless erosive arthritis. *Gut*.

[20] Mistilis S. P., Skyring A. P., Blackburn C. R. (1968). Natural history of active chronic hepatitis. I. Clinical features, course, diagnostic criteria, morbidity, mortality and survival. *Australasian Annals of Medicine*.

[21] Bouchier I. A., Rhodes K., Sherlock S. (1964). Serological abnormalities in patients with liver disease. *The British Medical Journal*.

[22] Clarke A. K., Galbraith R. M., Hamilton E. B. D., Williams R. (1978). Rheumatic disorders in primary biliary cirrhosis. *Annals of the Rheumatic Diseases*.

[23] Bearn A. G., Kunkel H. G., Slater R. J. (1956). The problem of chronic liver disease in young women. *The American Journal of Medicine*.

[24] Alpert E., Isselbacher K. J., Schur P. H. (1972). Pathogenesis of arthritis associated with viral hepatitis. *Medecine et Chirurgie Digestives*.

[25] Chen S.-Y., Chen C.-H., Huang Y.-C., Chan C.-J., Chen D.-C., Tsai F.-J. (2014). Genetic susceptibility to idiopathic membranous nephropathy in high-prevalence Area, Taiwan. *BioMedicine*.

[26] Chou L. W., Hsieh Y. L., Kuan T. S., Hong C. Z. (2014). Needling therapy for myofascial pain: recommended technique with multiple rapid needle insertion. *BioMedicine*.

[27] Zuckerman E., Yeshurun D., Rosner I. (2001). Management of hepatitis C virus-related arthritis. *BioDrugs*.

[28] Zuckerman E., Keren D., Rozenbaum M. (2000). Hepatitis C virus-related arthritis: characteristics and response to therapy with interferon alpha. *Clinical and Experimental Rheumatology*.

[29] Mueller S., Millonig G., Seitz H. K. (2009). Alcoholic liver disease and hepatitis C: a frequently underestimated combination. *World Journal of Gastroenterology*.

[30] Louie K. S., Micallef J. M., Pimenta J. M., Forssen U. M. (2011). Prevalence of thrombocytopenia among patients with chronic hepatitis C: a systematic review. *Journal of Viral Hepatitis*.

[31] Galossi A., Guarisco R., Bellis L., Puoti C. (2007). Extrahepatic manifestations of chronic HCV infection. *Journal of Gastrointestinal and Liver Diseases*.

[32] Wang Q.-Q. (2011). Analysis of the academic advantage of Huangdi Neijing based on future medical trends. *Journal of Chinese Integrative Medicine*.

